# The Mouse-Specific Splice Variant mRAGE_v4 Encodes a Membrane-Bound RAGE That Is Resistant to Shedding and Does Not Contribute to the Production of Soluble RAGE

**DOI:** 10.1371/journal.pone.0153832

**Published:** 2016-09-21

**Authors:** Stefania Di Maggio, Elena Gatti, Jaron Liu, Matteo Bertolotti, Günter Fritz, Marco E. Bianchi, Angela Raucci

**Affiliations:** 1 Experimental Cardio-Oncology and Cardiovascular Aging Unit, Centro Cardiologico Monzino-IRCCS, Milan, Italy; 2 Division of Genetics and Cell Biology, San Raffaele Scientific Institute, Milano, Italy; 3 Institute for Neuropathology, University of Freiburg, Freiburg, Germany; 4 Università Vita Salute San Raffaele, Milano, Italy; University of Illinois at Chicago, UNITED STATES

## Abstract

The receptor for advanced glycation end-products (RAGE) is involved in the onset and progression of several inflammatory diseases. The RAGE primary transcript undergoes numerous alternative splicing (AS) events, some of which are species-specific. Here, we characterize the mouse-specific mRAGE_v4 splice variant, which is conserved in rodents and absent in primates. mRAGE_v4 derives from exon 9 skipping and encodes a receptor (M-RAGE) that lacks 9 amino acids between the transmembrane and the immunoglobulin (Ig) domains. RNA-Seq data confirm that in mouse lung mRAGE_v4 is the most abundant RAGE mRNA isoform after mRAGE, which codes for full-length RAGE (FL-RAGE), while in heart all RAGE variants are almost undetectable. The proteins M-RAGE and FL-RAGE are roughly equally abundant in mouse lung. Contrary to FL-RAGE, M-RAGE is extremely resistant to shedding because it lacks the peptide motif recognized by both ADAM10 and MMP9, and does not contribute significantly to soluble cRAGE formation. Thus, a cassette exon in RAGE corresponds to a specific function of the RAGE protein–the ability to be shed. Given the differences in RAGE AS variants between rodents and humans, caution is due in the interpretation of results obtained in mouse models of RAGE-dependent human pathologies.

## Introduction

The receptor for advanced glycation end-products (RAGE) is a pattern recognition receptor (PRR) that binds numerous ligands, including advanced glycation end-products (AGE), several S100/calgranulins, amyloid-beta peptide, High Mobility Group Box 1 protein (HMGB1), the integrin Mac-1 and extracellular matrix components [1, 2]. In non-pathological conditions, RAGE is present in the lung at far greater levels compared to other tissues [3, 4]. RAGE expression is induced by its ligands and is associated to the progression of several acute and chronic inflammatory diseases [5–10].

Structurally the mature RAGE protein consists of three extracellular Ig-like domains, a single transmembrane helix and a short cytoplasmic tail [1]. The RAGE primary transcript undergoes numerous alternative splicing (AS) events in human, mouse and other species, which generate both conserved and species- and tissue-specific coding and non-coding mature transcripts [11–13]. The variants hRAGE and mRAGE, in human and mouse respectively, are the most abundant isoforms and encode the membrane-bound full-length protein (FL-RAGE) [11–13]. hRAGE_v1 in human, and mRAGE_v1/v3 in mouse, encode the secreted esRAGE protein, which represents a fraction of soluble RAGE (sRAGE); esRAGE has a unique C-terminal sequence [11, 12, 14, 15]. The variant mRAGE_v4 derives from exon 9 skipping, which results in the omission of 9 amino acids (ETGDEGPAE) in the extracellular portion of the receptor, close to the transmembrane domain. Although mRAGE_v4 is a prevalent mouse-specific RAGE variant [12], the corresponding protein has not been characterized yet.

We and others have demonstrated that soluble RAGE can also arise from the shedding of FL-RAGE ectodomain by proteases ADAM10 and MMPs [16–19]. Both in human and mouse, cleavage of FL-RAGE generates soluble cleaved RAGE (cRAGE) and a C-terminal fragment (CTF-RAGE) [17, 20]. Soluble RAGEs bind ligands with affinity similar to FL-RAGE, and prevent RAGE signaling by functioning as decoy receptors [17, 21–24]. Cross-sectional studies on human populations have suggested circulating sRAGEs as potential endogenous protective biomarkers in RAGE-mediated disorders [23, 25, 26].

Since the majority of the studies on RAGE functions have been performed in mouse models, exploring the differences in RAGE variants between humans and mice is fundamental in order to better understand the contribution of RAGE to human physiology and pathology.

Here, we characterize the mouse-specific mRAGE_v4 splice variant. We find that mRAGE_v4 corresponds to a membrane-bound protein (that we named M-RAGE) with a molecular weight lower than FL-RAGE; both isoforms are expressed at high level in the lung. Contrary to FL-RAGE, M-RAGE is resistant to proteolytic shedding and hence does not contribute to soluble cRAGE production, as it lacks the stretch of amino acids necessary for ADAM10 and MMP9 recognition. The transcript mRAGE-v4 is present in other rodents (*Rattus norvegicus*) and is absent in other primates (chimpanzee, rhesus macaque). Analysis of the cassette exon 9 in mouse *Ager* gene revealed splicing regulatory sequences present in rodents but not in primates.

Our results suggest that, given the differences in the biology of RAGE between rodents and humans, attention should be paid in the interpretation of results using mouse models for RAGE-dependent human pathologies.

## Materials and Methods

### RNA-Seq data

RNA-Seq data from murine heart and lung tissues were obtained from ENCODE portal (https://www.encodeproject.org/, released before June 2015). Experiments from the laboratories of Thomas Gingeras (CSHL) and Barbara Wold (Caltech) were chosen because of the larger number of already aligned reads files (BAM format) available in mm9 and mm10 assemblies. More than one BAM file is available for each experiment. We analyzed expression data from different life stages including embryonic (mm10), postnatal (mm10) and adult (mm9) tissues. ENCODE experiment ID for mouse: heart (ENCSR727FHP, ENCSR691OPQ, ENCSR510ADJ, ENCSR035DLJ, ENCSR000BYQ), lung (ENCSR039ADS, ENCSR000BYT). Cufflinks v2.2.0 [27] was used to estimate the relative abundances of each transcript of the mouse *Ager* gene. Ensembl transcripts (release v67 and v79 for mm9 and mm10 respectively) for mouse *Ager* gene were provided by a GTF file using default parameters. The expression of each transcript was measured in Fragments Per Kilobase of exon model per Million mapped fragments (FPKM) and represented in a heatmap using R functions (http://www.R-project.org).

### Comparative sequence analysis

Identification of splice junctions was carried out comparing the mRNA and genomic Ager sequences in in *Mus musculus* (mm10, NM_007425), *Homo sapiens* (hg19, NM_001206929), *Rattus norvegicus* (rno5, NM_053336), *Pan troglodytes* (*panTro4*, XM_009450977.1) *and Macaca mulatta* (*rheMac3*, NM_001205117). Sequence similarity at mRNA and protein level was determined using BLASTn and BLASTp (http://www.ncbi.nlm.nih.gov/BLAST).

The web-based ESEfinder 2.0 program [28] (http://rulai.cshl.edu/tools/ESE/) was used to search for all exonic splicing enhancer (ESE) motifs. Input sequences (around exon 9 of the mouse, rat and human *Ager* genes) were screened for all possible hexamers, heptamers and octamers previously identified as putative SR-binding sites using any of the five available matrices in the ESEfinder web server (SF2/ASF, SF2/ASF BRCA1, SC35, SRp40 and SRp55) using default threshold (1.956 for SF2/ASF, 1.867 for SF/ASF BRCA1, 2.383 for SC35, 2.670 for SRp40, 2.676 for SRp55). The program scores the input sequences according to their fit to loose consensus sequences; scores above a default threshold value are predicted as SR protein binding sites and thus to function as ESEs.

When overlapping putative binding sites of the same SR protein occurs, we keep only one ESE for the same hexamer (or heptamer or octamer). In mouse exon 9 we detect 2 couples of overlapping heptamers (ESR A and ESR B).

### Molecular modeling

Molecular models of RAGE proteins was performed with the program Modeller [29]. Domains were oriented with Coot [30] and figures were prepared with PyMol (The PyMOL Molecular Graphics System, Version 1.7.4 Schrödinger, LLC).

### Animals

Studies on *Rage*-/- [22] and C57BL/6 wild type mice (Charles River Laboratories, Calco, Italy) were conducted according to a protocol approved by the San Raffaele Scientific Institute Animal Use Committee, and adhered to the Italian Ministry of Health guidelines. Mice were housed in standard cages on a 12:12 h light-dark cycle and fed a normal chow diet ad libitum. Three month-old animals were euthanized by CO_2_ inhalation; lungs were dissected out, washed in cold PBS, kept at -20°C and eventually processed as described below.

### Antibodies

Anti-β-actin monoclonal Ab was purchased from Sigma-Aldrich (1 μg/ml; cat. A2228; St. Louis, MO, USA), goat anti-human RAGE (α-RAGE N-term1; recognizes mouse and rat RAGE as well) from R&D Systems (cat. AF1145; Minneapolis, MN, USA) was used for western blot (WB; 1 μg/ml) and Immunofluorescence (IF; 4 μg/ml) and rabbit anti-human RAGE (α-RAGE N-term2; recognizes mouse and rat RAGE as well) from Santa Cruz Biotechnology (0.4 μg/ml; cat. Sc-5563; Santa Cruz, CA, USA) was used for WB. Rabbit polyclonal antibody against the intracellular domain of human/mouse RAGE was from Abcam (α-RAGE C-term; 1 μg/ml; cat. ab3611-100; Cambridge, UK). Anti-GAPDH was from Santa Cruz Biotechnology (0.4 μg/ml; cat. sc-468).

### Cell culture

Immortalized MEFs from *Adam10*−/− (A10 ko) and *Adam10*+/+ (A10 wt) mice were described elsewhere [17, 31] MEFs and R3/1 cells [2] were grown in Dulbecco’s modified Eagle medium (DMEM) supplemented with 10% fetal calf serum (FCS) and 1% penicillin/streptomycin.

### Plasmids

We generated the plasmids expressing mRAGE or mRAGE_v4 cDNA under the CMV promoter by cloning into *Eco*RI sites of the pcDNA3 vector (pcDNA/FL-RAGE and pcDNA/M-RAGE, respectively). The two cassettes representing mouse RAGE splice variants were produced as previously described [12]. Briefly, a PCR was performed on the murine lung cDNA, isolated from a C57BL/6 wild type mice, using the forward mouse RAGE 5’-UTR [5’-AGGAAGCACCATGCCAGC-3’] and the reverse mouse RAGE 3’-UTR [5’-GGATGGAATGTGGGGGAG-3’] primers with the Phusion High-Fidelity DNA polymerase system (Finnzymes, Massachusetts, USA). The resulting PCR product was purified using the Qiaquick PCR Clean-Up kit (Qiagen, Hilden, Germany) and subsequently cloned into the TOPO TA vector pCR2.1-TOPO (Invitrogen, California, USA) after addition of 3’ A overhangs using Taq polymerase (Finnzymes). Plasmid DNA was purified from 24 randomly selected bacterial colonies, sequenced using T7 and M13R primers, and the confirmed splice variants were then subcloned.

### Western blot (WB) and immunofluorescence (IF)

Lungs isolated from *Rage*-/- (N = 1) and wild type C57BL/6 (N = 3) mice were lysed in RIPA buffer (10 mM Tris-HCl, pH 7.2; 150 mM NaCl; 5 mM EDTA; 0.1% SDS; 1% sodium deoxycholate; 1% Triton X-100) in the presence of protease inhibitors (1:100 Sigma protease inhibitor cocktail). After 5 min on ice, samples were centrifuged at 10,000 g for 30 min at 4°C. Supernatants were recovered, and protein concentration was determined by the Bradford method (Bio-Rad, Munich, Germany), using BSA as a standard. Laemmli buffer was added to soluble cell lysates and samples were heated for 5 mins at 95°C before loading on sodium dodecyl sulfate (SDS) polyacrylamide protein gels (10%).

For detection of soluble RAGE in supernatants, R3/1 cells or MEFs (1x10^5^) were seeded in 6-cm tissue culture plates and transfected with the indicated plasmids using Fugene HD (Promega, WI, USA) according to the manufacturer’s indications. After 48 hours, the complete medium was replaced with OPTIMEM medium for 5 (R3/1 cells) or 3 (MEFs) hours and incubated at 37°C. Where indicated, transfected R3/1 cells and MEFs were treated with ADAM10 inhibitor (GI254023X, abbreviated here as GI, 5 μM; Cat. SML0789; Sigma-Aldrich) or PMA (100 nM; Sigma-Aldrich), respectively. Culture supernatants were centrifuged and then precipitated with half a volume of 100% cold acetone and dissolved in 2X Laemmli buffer. Aliquotes of supernatant were tested for cRAGE by ELISA assay. Cells were washed with PBS and lysed in RIPA buffer in the presence of protease inhibitors, and processed for testing RAGE expression with the indicated antibodies as already described [2, 17]. Bands on films were quantified using ImageJ software (U.S. National Institutes of Health, Bethesda, MD, USA).

For IF, R3/1 cells were transfected with the indicated plasmids using Fugene HD and processed as previously reported [2]. Images were acquired on a Leica TCS SP5 AOBS confocal microscope equipped with a ArgonPlus Ar-ion laser and a HCX PL Apo CS 63x oil immersion objective/1.4 NA.

### ELISA assay

Total mouse cRAGE in culture supernatant was determined using a Quantikine ELISA kit specific for mouse RAGE (cat. MRG00; R&D Systems), according to the manufacturer’s protocol. The minimum detectable dose of soluble RAGE was 0.031 ng/ml, and linearity was conserved between 0.031 and 2 ng/ml.

### Data analysis

Results are expressed as mean ± SD. Statistical analysis was performed by one-way ANOVA with post hoc Bonferroni’s multiple comparisons test using GraphPad Prism program. Values of P < 0.05 were considered significant.

## Results

### Expression patterns of alternatively spliced mRNA isoforms in the mouse

**Fig 1:**
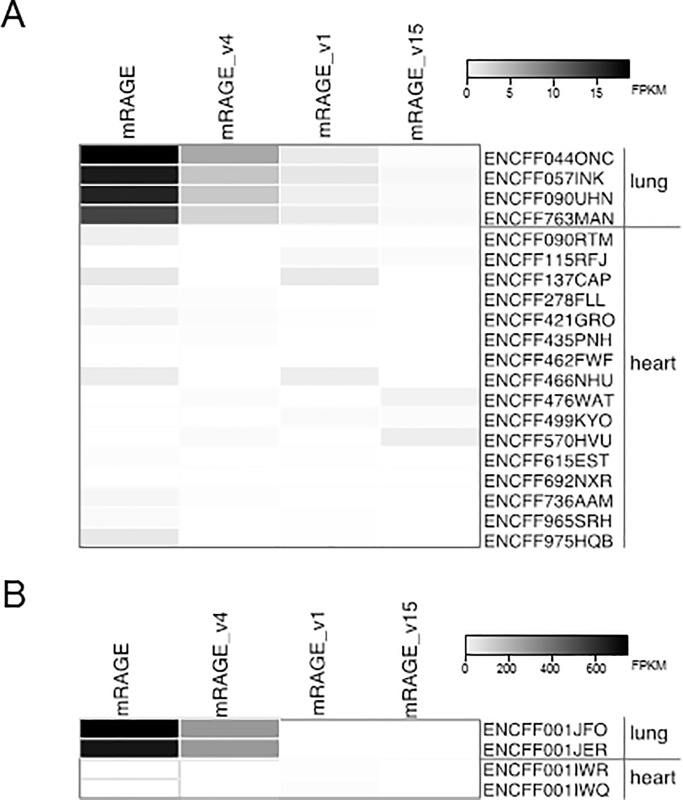
Expression of RAGE transcript variants in different life stages of *Mus musculus*. The heatmap shows the expression level of indicated transcripts of murine *Ager* gene in lung and heart tissues in embryonic/postnatal (A) and adult samples (B). Scale varies from white (no expression) to black (highest expression).

We explored by RNA-Seq analysis the relative expression of all coding RAGE splice variants across embryonic, post-natal and adult mouse heart and lung samples. We confirmed that multiple alternatively spliced RAGE mRNA isoforms (mRAGE, mRAGE_v4, mRAGE_v1, mRAGE_v15) exist in the mouse at all the considered life stages. Expression differs between heart and lung [12] (Fig 1). It is not possible to define the relative amount for each RAGE splice variant in both embryonic/postnatal (Fig 1A) andadult (Fig 1B) hearts because the expression is weak (FPKM < 6) and only detectable with low confidence for some samples. By contrast, the level of RAGE splice isoforms is remarkably high in embryonic/postnatal lungs (Fig 1A) and even more abundant in adult lungs (Fig 1B). All the analyzed mouse lung samples show a similar pattern of expression. In particular, the most prevalent pulmonary isoform is mRAGE and the second major one is mRAGE_v4. mRAGE_v1 has low but uniform expression among the samples, while mRAGE_v15 is under the detection limit (Fig 1).

### Murine RAGE_v4 transcript encodes a membrane-bound RAGE resistant to shedding

**Fig 2:**
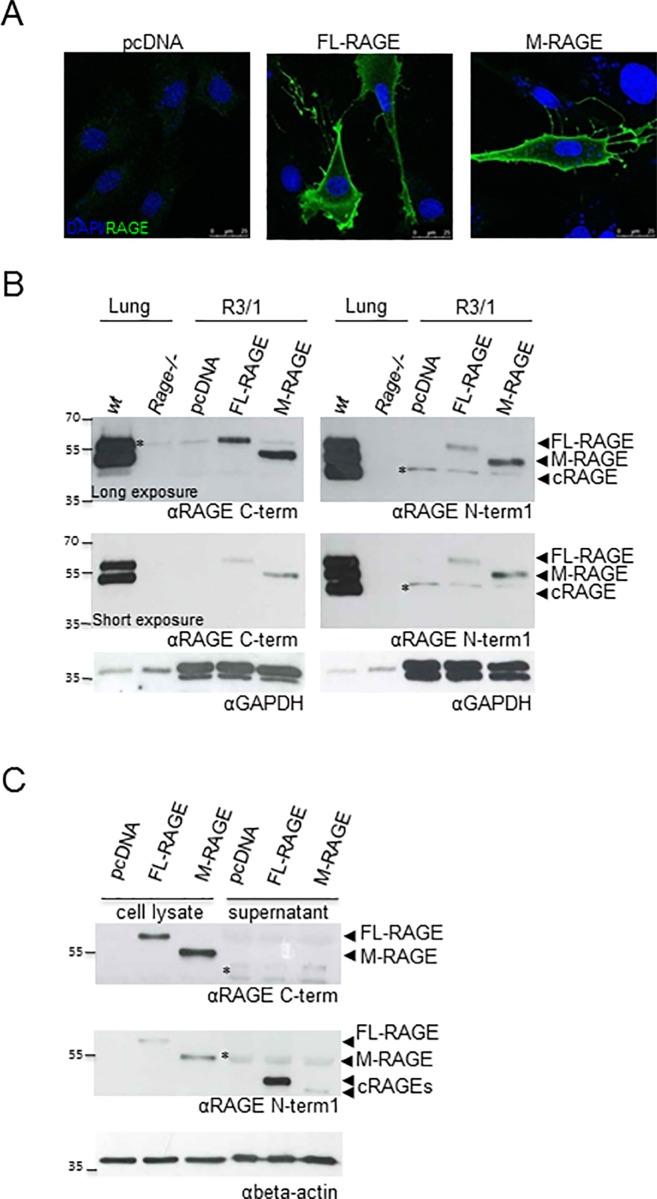
M-RAGE is highly resistant to shedding. **(A)** Cellular localization of FL-RAGE and M-RAGE. R3/1 cells were transfected with the empty vector (pcDNA), FL-RAGE or M-RAGE and IF staining was performed using the RAGE N-term antibody (green) that recognizes the extracellular domain. Nuclei were stained with DAPI (blue). Bars correspond to 25 μm. **(B)** FL-RAGE and M-RAGE are readily detectable in mouse lung. Representative WB of twenty μg of lysate from one *Rage-/-* and one wild type (wt) lung and 35 μg of lysate from R3/1 cells transfected with the empty vector (pcDNA), FL-RAGE or M-RAGE were probed with the indicated antibodies against RAGE. Because of different levels of RAGE expression in lung and R3/1 cells, long and short exposures of the same membranes are shown. GAPDH was used as loading control; (N = 1 *Rage*-/- mouse; N = 3 wild type C57BL/6 mice). **(C)** M-RAGE is not shed from cultured cells. R3/1 cells were transfected with pcDNA, FL-RAGE or M-RAGE, and cell lysates (35 μg) or supernatants were analyzed with the indicated antibodies against RAGE. Beta-actin was used as loading control. In all panels, nonspecific bands (*) are indicated.

We then characterized mRAGE_v4 at the protein level. mRAGE codes for the mature membrane-bound full length receptor, while mRAGE-v4 for a similar protein lacking 9 amino acid in the linker region connecting the C2 domain with the transmembrane helix. We generated plasmids expressing cDNAs corresponding to mRAGE and mRAGE_v4, and transiently transfected them, or the empty vector (pcDNA) as control, into rat alveolar type I-like R3/1 cells, which do not express detectable amounts of endogenous RAGE [2]. As expected, both protein isoforms were localized on the cell membrane (Fig 2A). Cells transfected with mRAGE cDNA showed in WBs a band of about 58 KDa, and cells transfected with mRAGE-v4 cDNA a band of about 50 KDa, recognized by antibodies against the intracellular (α-RAGE C-term) or the extracellular (α-RAGE N-term1) domains of RAGE (Fig 2B; S1 Fig). We named these two isoforms FL-RAGE and membrane RAGE (M-RAGE), respectively. Both proteins are expressed at high and almost equal level in the mouse lung, and they correspond to the two highest mw protein bands recognized by both α-RAGE antibodies (Fig 2B; S1 Fig). The third form of RAGE present in the lung is the soluble cRAGE that derives from the shedding of FL-RAGE; cRAGE is recognized by α-RAGE N-term1 but not by α-RAGE C-term antibodies (Fig 2B; S1 Fig) [17].

Since analysis of mouse lung lysates revealed only one cRAGE band, we hypothesized that M-RAGE could not be proteolytically processed. Thus, we tested whether M-RAGE could be subject to shedding. FL-RAGE and M-RAGE were detected in the corresponding cell lysates both with α-RAGE C-term and α-RAGE N-term1 antibodies. Supernatants of R3/1/FL-RAGE, but not R3/1/pcDNA cells, contained soluble cRAGE, detectable only with α-RAGE N-term1 antibody (Fig 2C; S1 Fig). Interestingly, supernatants of R3/1/M-RAGE cells exhibit an extremely low amount of cRAGE with a lower molecular weight.

These data demonstrate that, *in vivo* pulmonary M-RAGE is not cleaved and that soluble cRAGE derives from FL-RAGE shedding, while, *in vitro*, just a small fraction of M-RAGE undergoes proteolysis.

### M-RAGE lacks the MMP9 consensus sequence and the cleavage site for ADAM10

**Fig 3:**
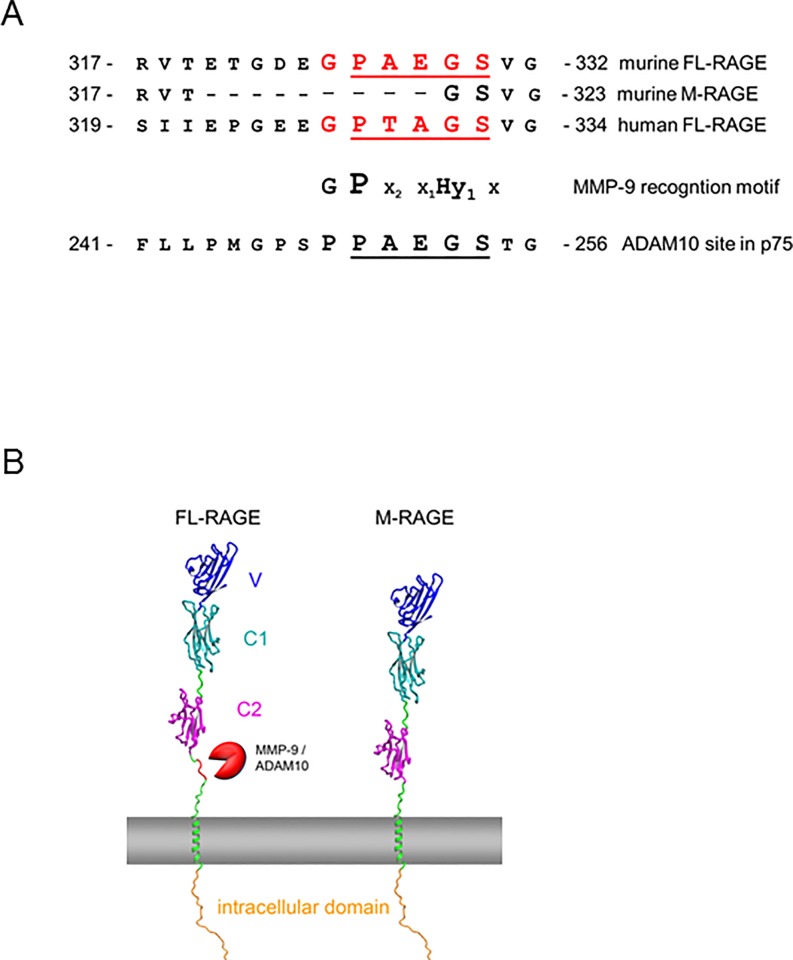
M-RAGE lacks a protease recognition site. **(A)** Sequence alignment of murine FL-RAGE and M-RAGE with human FL-RAGE. M-RAGE lacks nine amino acid residues relative to FL-RAGE. This stretch of residues comprises an MMP-9 recognition site (red) that overlaps with a motif recognized by ADAM10 protease (underlined). **(B)** Three-dimensional models of FL-RAGE and M-RAGE. The three Ig domains of RAGE (V, C1, and C2) are shown in different colors, the transmembrane helix is shown in green and the location of the membrane is indicated in grey. The protease recognition site in FL-RAGE is highlighted in red. Clearly, in FL-RAGE the linker between C2 and the transmembrane helix is extended and more easily accessible by proteases such as MMP-9 or ADAM10.

To explore why M-RAGE is resistant to proteolysis, we analyzed the RAGE protein sequence for the presence of putative protease recognition sites. As shown previously by our laboratory and other research groups, human RAGE is cleaved by ADAM10 and MMP9 [16, 17, 19]. MMP9 recognizes the consensus motif X_4_-P_3_-X_2_-X_1_-Hy_1’_, where X_4_ is often a glycine and Hy is a hydrophobic residue and cleavage occurs between X_1_ and Hy_1’_. Interestingly, FL-RAGE contains a MMP9 consensus motif G_4_-P_3_-A_2_-E_1_*-G_1’_ in the linker region connecting C2 domain with the transmembrane helix (residues 325 to 329, the star indicates the bond that is cleaved). This motif is conserved in human RAGE, with G_4_-P_3_-T_2_-A_1_*-G_1’_ as residues 327–331. Notably, M-RAGE lacks precisely this motif (Fig 3A).

The prediction of the ADAM10 cleavage site in RAGE is not straightforward. In contrast to MMP9 there is no clear consensus motif recognized by ADAM10 [32]. Nevertheless, comparison with bona fide ADAM10 substrates revealed that FL-RAGE contains the sequence P_4_-A_3_-E_2_-G_1_*-S_1’,_ which is identical to the ADAM10 cleavage site in the p75 TNF receptor [32] (Fig 3A). Notably, soluble RAGE isolated from murine lung ends at the C-terminus with Glu 328 [20]. Incidentally, this suggests that in mice RAGE shedding occurs predominantly via ADAM10, since the C-terminus produced by MMP9 proteolysis should end with Gly 329.

In order to evaluate the accessibility of the recognition site by the proteases we generated a structural model of murine FL-RAGE and M-RAGE. In both proteins the C2 domain is connected to the transmembrane helix by a linker region that is predicted to contain no secondary structure (Fig 3B). The linker comprises 18 residues in FL-RAGE whereas it is shortened to 9 residues in M-RAGE. MMP9 binds its substrates via an extended stretch of at least 9 amino acid residues (Fig 3B). Our model suggests that the shortening of the linker region in M-RAGE not only eliminates the putative recognition site for MMP9 and ADAM10 but also reduces the steric accessibility for the proteases.

### Shedding of FL-RAGE but not of M-RAGE is enhanced by PMA-induced ADAM10 activity

**Fig 4:**
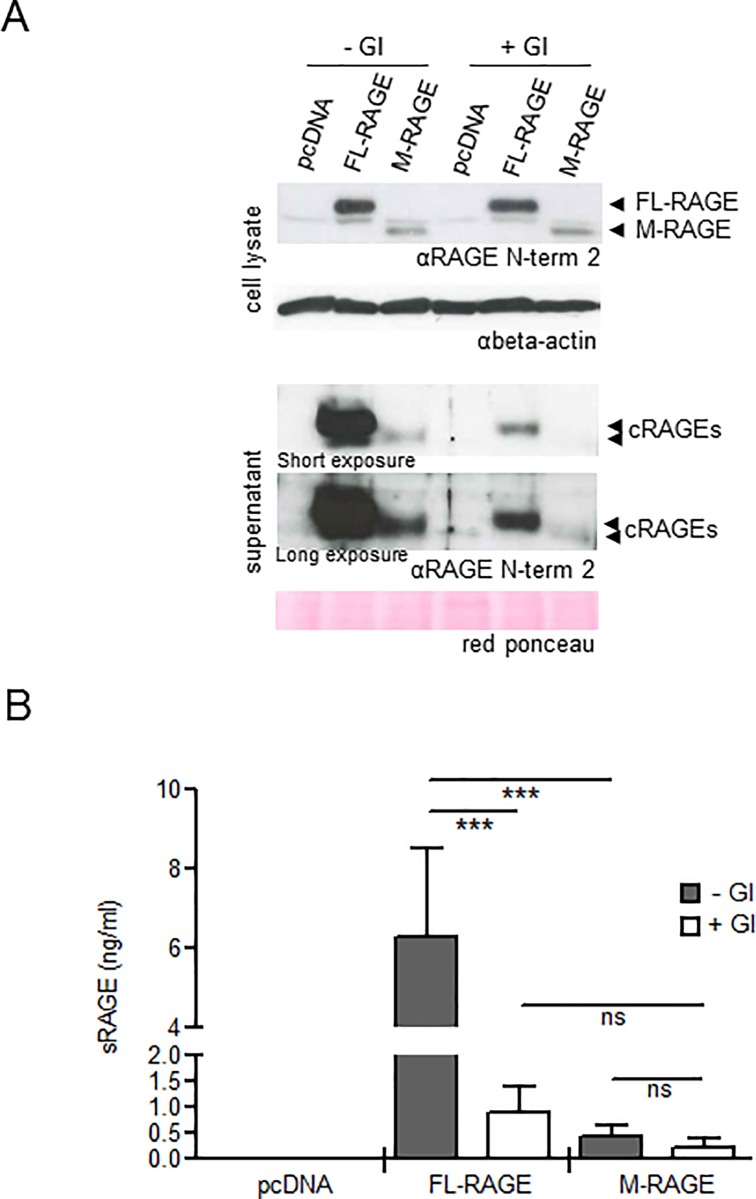
Effect of ADAM10 activity on FL-RAGE and M-RAGE cleavage. **(A, B)** R3/1 cells were transfected with the indicated vectors, treated or not with an ADAM10 inhibitor (GI), and processed as described in Materials and Methods. (A) Representative WB of cell lysates (50 μg) and supernatants of R3/1 cells. Because of the variable levels of cRAGEs in the supernatants of R3/1 cells, short and long exposures of the same membrane are shown. Beta-actin and red Ponceau staining were used as loading control for cell lysates and supernatants, respectively. (B) Quantification of cRAGEs in the supernatant of transfected R3/1 cells by ELISA assay. R3/1 cells were transfected with different efficiency and the volume of supernatants tested was adjusted accordingly. Data are mean ± SD (N = 3; ***, P≤0.0001; ns, not significant; a.u., arbitrary units).

**Fig 5:**
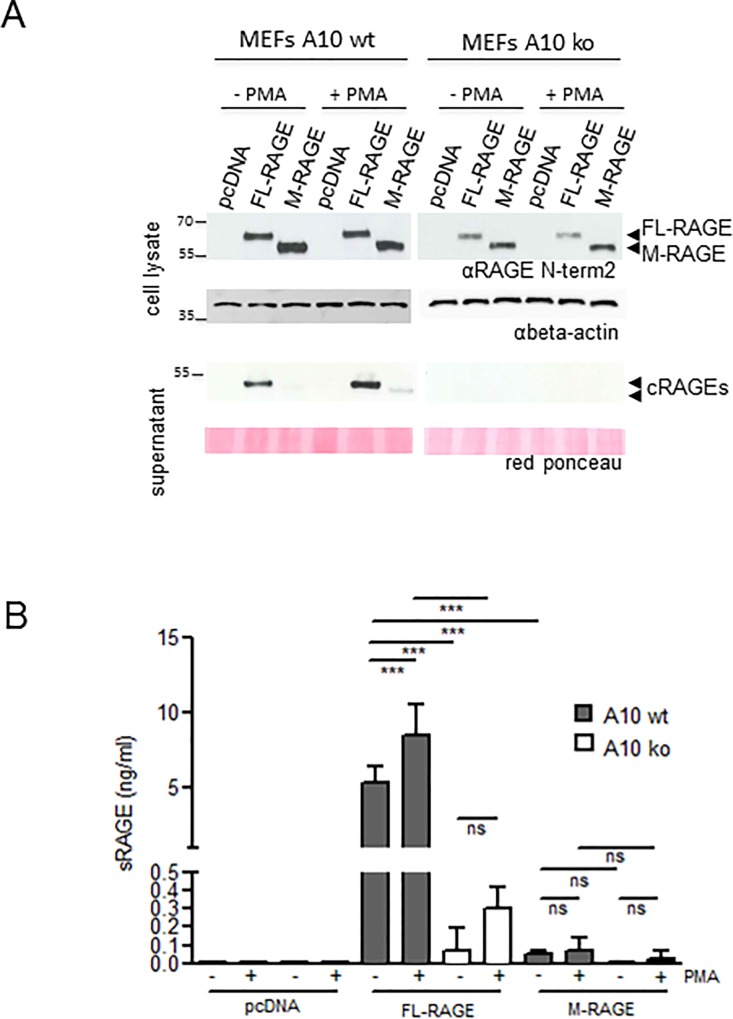
PMA enhances FL-RAGE but not M-RAGE shedding. **(A, B)** A10 ko and A10 wt MEFs were transfected with the indicated vectors, treated or not with PMA for 3 hours and processed as described in Materials and Methods. (A) Representative WB of cell lysates (40 μg) and supernatants of MEFs. Beta-actin and red Ponceau staining were used as loading control for cell lysate and supernatant, respectively. (B) Quantification of cRAGEs in the supernatants of transfected MEFs by ELISA assay. MEFs cells were transfected with different efficiency and the volume of supernatant tested was adjusted to compensate. Data are mean ± SD (N = 3; ***, P≤0.0001; a.u., arbitrary units).

Since ADAM10 is likely to be the major sheddase for murine FL-RAGE in the lung [20], we sought to determine experimentally whether murine FL-RAGE and M-RAGE are ADAM10 targets. We first treated or not R3/1/pcDNA, R3/1/FL-RAGE and R3/1/M-RAGE cells with the GI compound, a specific inhibitor of ADAM10 activity. Cell lysates and supernatants of cells were analyzed for the expression of RAGE isoforms by WB (Fig 4A; S2 Fig). Since we observed a difference in the efficacy of transfection of R3/1 cells with FL-RAGE and M-RAGE vectors, cRAGE amount was quantified in the supernatant of cells after adjusting for total RAGE expression by ELISA (Fig 4B). GI inhibited constitutive shedding of murine FL-RAGE, reducing the levels of the corresponding cRAGE close to those of cRAGE derived from M-RAGE cleavage (Fig 4; S2 Fig). Notably, GI treatment further reduced the small amount of cRAGE deriving from M-RAGE proteolysis, although the difference was not statistically significant (Fig 4B). These data were confirmed using *Adam10*-/- (A10 ko) MEFs and the corresponding *Adam10*+/+ (A10 wt) MEFs (Fig 5; S3 Fig).

PMA is a known activator of ADAM10 [31] and we have previously shown that human FL-RAGE shedding is induced by PMA stimulation in human cell lines [17]. We treated transfected R3/1 cells or MEFs with PMA. PMA treatment of R3/1 cells did not influence the cleavage of both FL-RAGE and M-RAGE (not shown). A10 wt MEFs stimulated with PMA exhibit an enhanced shedding of FL-RAGE, whereas PMA-treated A10 ko MEFs did not show an equivalent increase in shedding (Fig 5; S3 Fig). PMA did not significantly affect M-RAGE shedding in either A10 wt and A10 ko MEFs (Fig 5; S3 Fig).

Altogether, these data indicate that murine FL-RAGE is shed mostly by ADAM10, as efficiently as human FL-RAGE, and that *in vitro* ADAM10 constitutively cleaves a small fraction of M-RAGE, compatible with the absence of its consensus sequence. However, when ADAM10 activity is induced, M-RAGE proteolysis is not enhanced confirming its resistance to ADAM10-dependent shedding.

### Sequence features of the mRNA generating M-RAGE

We performed further cross-species comparisons using both mRNA and the protein sequence. BLASTn and BLASTp results reveal orthologs of mRAGE-v4 only in rodents (*Rattus norvegicus*, advanced glycosylation end product-specific receptor variant 3). In terms of exon/intron structure, no equivalent splice variant was found in human or in other primates (chimp, rhesus; not shown), as already reported [13].

**Fig 6:**
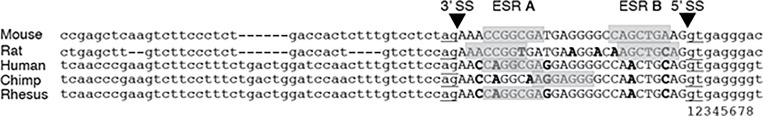
Splicing sequence features in orthologous Ager cassette exon 9. Alignment of exon 9 of the *AGER* gene among mouse, rat, human, chimp and rhesus. Upper and lower case indicate exonic and intronic sequence, respectively. The AG and GT of the 3’SS and 5’SS are underlined. The examined ESR A and B sites are highlighted in gray. Nucleotides in bold indicate exonic positions that change during evolution.

With the aim to understand the mechanism by which exon 9 is sometimes skipped in rodents but never in primates, we examined the sequences of the primary RAGE transcript around cassette exon 9 (Fig 6). The 5’ splicing sites (SS) in all the analyzed mammals have the obligatory GT at positions 1 and 2, are canonical and differ among each other only for the 8^th^ nucleotide. The 5’ss of mouse has the weakest score (9.16) relative to the other mammals, but the rat 5’ ss has the same score as primates (10.07), indicating that the 5’ ss does not justify the difference in splicing between rodents and primates. Regarding the 3’ ss, mouse and rat have scores (8.23 and 7.42 respectively) similar to those of primates (8.37), which again do not justify the splicing differences.

We then examined the sequence features outside the canonical splice sites. We analyzed the distribution of putative exonic splicing regulators (ESRs), sequences that act as binding sites for members of the serine/arginine rich family of splicing enhancer proteins [33, 34] Using ESEfinder [28] we identified 4 putative ESRs in mouse. Interestingly, ESR B was found in mouse and rat, but not in the other mammals (Fig 6).

## Discussion

AS is a common mechanism in eukaryotic organisms to expand transcriptomic and proteomic complexity through production of multiple transcripts and corresponding proteins from a single gene [35]. AS is involved in genomic evolution and in development, and its misregulation may directly cause or contribute to the severity of a given diseases [36].

RAGE has multiple tissue- and species-specific AS isoforms [11, 13, 37]. Major differences between human and murine RAGE splice transcripts and abundance have been reported [11–13, 36]. We were most interested in the mouse-specific mRAGE_v4 variant, which encodes a membrane-bound RAGE protein lacking 9 amino acids in the linker region between the extracellular Ig domains and the transmembrane domain. mRAGE_v4 or equivalent AS forms are not present in human. In agreement with previous published data [12, 13] our RNA-seq analysis revealed that among the mature transcripts, mRAGE_v4 is the second most represented splice variant in lung after mRAGE (Fig 1) [12, 13]; mRAGE_v1 and mRAGE_v15 were also found but at very low levels. It was not possible to define the relative amount of RAGE splice transcripts in heart because of their extremely low expression (Fig 1). The dissimilarities between lung and heart are not surprising, since in physiological conditions RAGE is present at high and detectable levels only in lung [3, 4].

We named the protein product of mRAGE_v4 membrane RAGE, M-RAGE, to distinguish from the mature full-length receptor FL-RAGE, which is encoded by the mRAGE transcript. As expected, M-RAGE localizes on the cellular surface and has an apparent MW of about 50 kDa, lower than that of FL-RAGE (58 kDa; Fig 2A and 2B; S1 Fig). Peng et al. have reported that while FL-RAGE is localized to the plasma membrane as well as to the early and late endosome, M-RAGE is exclusively localized to the plasma membrane [38]. Both proteins are highly and almost equally expressed in the murine lung, together with the soluble cRAGE (Fig 2B; S1 Fig). Similar to its human counterpart, murine FL-RAGE undergoes constitutive ectodomain shedding in R3/1 cells and MEFs, which depends mostly on ADAM10 activity (Figs 2C, 4 and 5; S1–S3 Figs). Interestingly, only a small fraction of M-RAGE experiences shedding *in vitro*. Correspondingly, M-RAGE is resistant to proteolysis and does not contribute significantly to cRAGE production in the lung (Figs 2C, 4 and 5; S1–S3 Figs). Indeed we and others have detected only one species of cRAGE in the pulmonary lysates (Fig 2B; S1 Fig) [20]. M-RAGE resistance to shedding can also explain the discrepancy between the relative amount of mRAGE and mRAGE_v4 transcripts and the corresponding proteins in the lung: it is likely that the constitutive FL-RAGE cleavage reduces its cell surface expression, while M-RAGE can accumulate.

Our structural analysis further suggests that the absence of the 9 amino acids in M-RAGE is unlikely to affect its ligand binding or adhesive properties. However, the missing amino acid stretch in M-RAGE contains the MMP9 consensus motif and the ADAM10 cleavage site, predicted on the base of its similarity to the cleavage site of the p75 TNF receptor, a bona fide ADAM10 substrate [32] (Fig 3A). Notably, cRAGE isolated from murine lung ends at the C-terminus with Gly 329 [20], suggesting that ADAM10, and not MMP9, is the major pulmonary protease responsible for FL-RAGE cleavage, at least in physiological conditions. Despite the absence of the predicted cleavage site, ADAM10 is still responsible for the small fraction of M-RAGE cleavage in R3/1 cells and MEFs, likely because of the overexpression of the receptor after transfection (Figs 4 and 5; S2 and S3 Figs). However, when ADAM10 activity is induced by PMA, FL-RAGE but not M-RAGE proteolysis is further enhanced, confirming experimentally that the lack of the peptide motif recognized by ADAM10 in M-RAGE is responsible for its resistance to shedding (Fig 5; S3 Fig).

We also investigated the exon–intron structure of the mouse *Ager* gene and found a high level of conservation of splicing regulatory elements in rat but not in human and other primates suggesting that those sites may regulate the AS of the exon 9 (Fig 6).

Species-specific variation in AS is an important engine in the evolution of mammalian species. At least 11% of human and mouse cassette exons undergo species-specific AS [39]. Remarkably, species-specific AS events disproportionately affect conserved sequences that dictate the functional properties of proteins [39]. mRAGE_v4 may represent one such example: indeed, the AS of exon 9 involves the deletion of a specific amino acid stretch indispensable for RAGE ectodomain shedding and cRAGE production.

Thus, our characterization of M-RAGE highlights important divergences in the biology of mouse and human RAGE that needs to be carefully considered since the mouse is the most extensively used animal model to study RAGE contribution to human pathogenesis.

For instance, RAGE is expressed at high level mainly on the membrane of alveolar type I (ATI) cells regulating proper lung development [40, 41]. Evidences from mouse studies support a pathogenic role of RAGE in pulmonary inflammatory disorders, in which RAGE isoforms, in particular FL-RAGE and cRAGE/sRAGE, have been found either increased or decreased [3, 4, 41]. Not much attention has been paid to M-RAGE, which we demonstrated here to be highly expressed in murine lung.

In the light of our findings, we believe that M-RAGE expression and regulation should be taken in consideration when investigating RAGE physiological and pathological functions. First, the ectodomain cleavage of adhesion molecules has been shown to regulate not only their membrane expression but also cell migration, adhesion and activation [42–44]; in this context, uncleavable M-RAGE may exert adhesive and migratory properties different from FL-RAGE. Second, we propose that, since M-RAGE does not participate to sRAGE production, it may contribute to explain the low levels of circulating murine sRAGEs. Human sRAGEs have elicited a lot of interest because their potential role as both a RAGE antagonist to prevent ligand-dependent receptor activation and circulating biomarkers for RAGE-mediated disorders [21–26]. Interestingly, analysis of murine serum/plasma has revealed no detectable soluble RAGE isoforms within the range seen in human [12]. Accordingly, the presence of AS variants encoding for esRAGE and soluble RAGEs in general is low in most tissues compared to human [12, 13]. The existence of M-RAGE may contribute to explain these differences.

In conclusions, the variation in RAGE AS between rodents and humans may have a profound evolutionary and functional meaning that needs to be further explored.

## Supporting Information

S1 Fig Original unadjusted blots of Fig 2: M-RAGE is resistant to shedding. Twenty μg of lysate from one *Rage-/-* and one wild type (wt) lungs and 35 μg of lysate or supernatants from R3/1 cells transfected with the empty vector (pcDNA), FL-RAGE or M-RAGE were probed with the indicated antibodies against RAGE. Because of different levels of RAGE expression in lung and R3/1 cells, long and short exposure of the same membranes are shown. GAPDH or Beta-actin were used as loading control. In all panels, nonspecific bands (*) are indicated.
(TIF)

S2 Fig Original unadjusted blots of Fig 4: effect of ADAM10 activity on FL-RAGE and M-RAGE cleavage. R3/1 cells were transfected with the indicated vectors, treated or not with an ADAM10 inhibitor (GI), and processed as described in Materials and Methods. Representative WB of cell lysates (50 μg) and supernatants of R3/1 cells. Because of the variable levels of cRAGEs in the supernatants of R3/1 cells, short and long exposures of the same membrane are shown. Beta-actin and red Ponceau staining were used as loading control for cell lysates and supernatants, respectively.
(TIF)

S3 Fig Original unadjusted blots of Fig 5: PMA enhances FL-RAGE but not M-RAGE shedding. A10 ko and A10 wt MEFs were transfected with the indicated vectors, treated or not with PMA for 3 hours and processed as described in Materials and Methods. Representative WB of cell lysates (40 μg) and supernatants of MEFs. Beta-actin and red Ponceau staining were used as loading control for cell lysate and supernatant, respectively.
(TIF)
