# Supplementary figures and images for: The Mouse-Specific Splice Variant mRAGE_v4 Encodes a Membrane-Bound RAGE That Is Resistant to Shedding and Does Not Contribute to the Production of Soluble RAGE
Source: PLoS One. 2016 Sep 21;11(9):e0153832. doi: 10.1371/journal.pone.0153832 (PMC5031469; doi:10.1371/journal.pone.0153832)

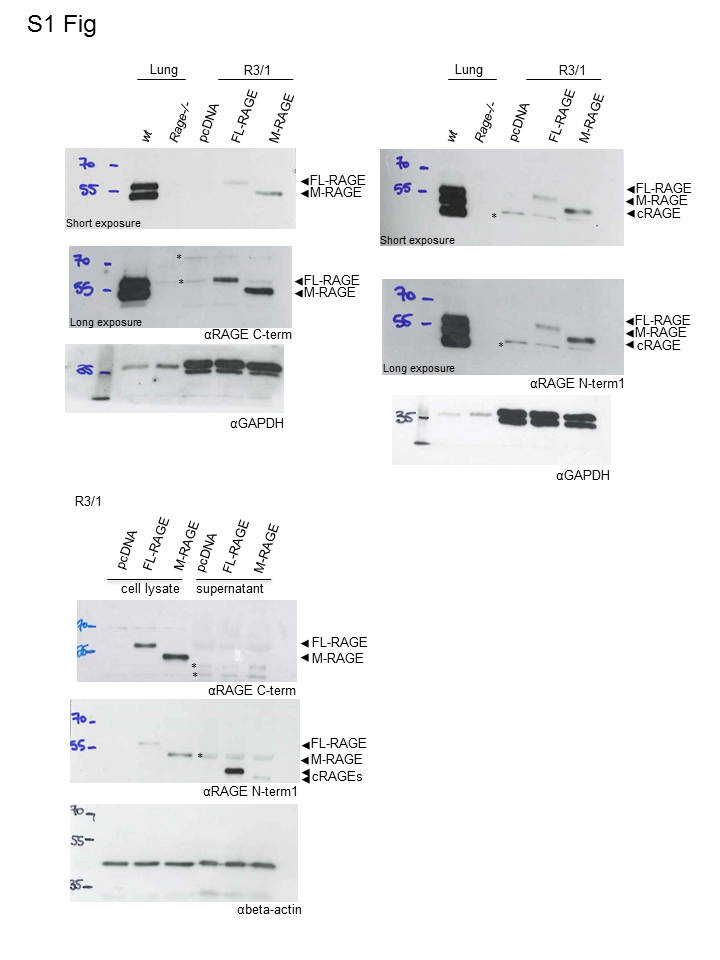

Supplement: S1 Fig — Twenty μg of lysate from one Rage-/- and one wild type (wt) lungs and 35 μg of lysate or supernatants from R3/1 cells transfected with the empty vector (pcDNA), FL-RAGE or M-RAGE were probed with the indicated antibodies against RAGE. Because of different levels of RAGE expression in lung and R3/1 cells, long and short exposure of the same membranes are shown. GAPDH or Beta-actin were used as loading control. In all panels, nonspecific bands (*) are indicated. (TIF) [file pone.0153832.s001.tif]

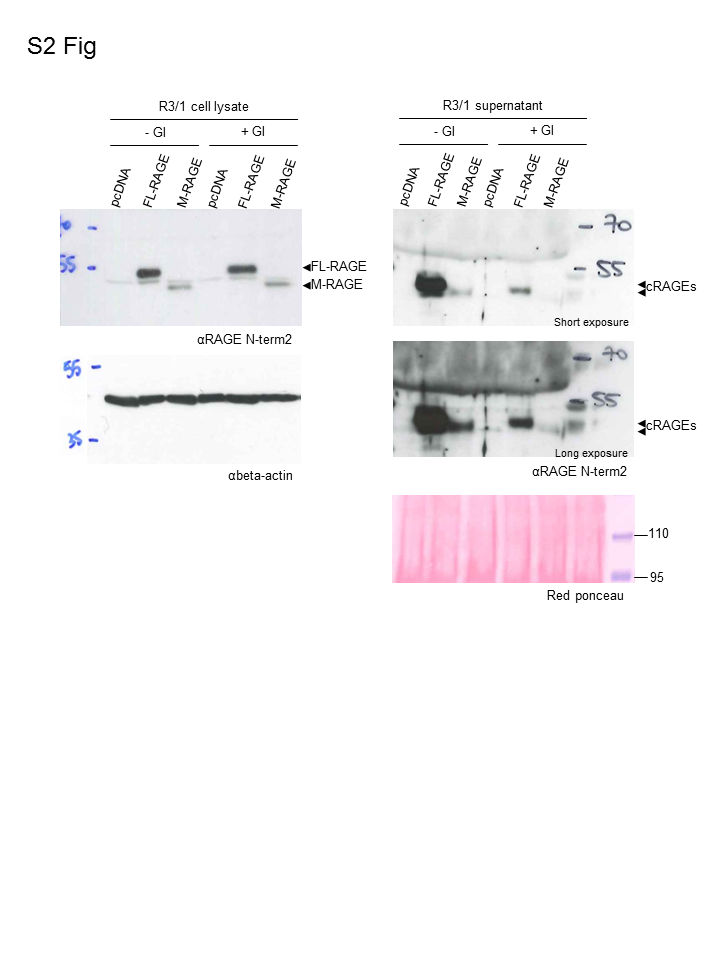

Supplement: S2 Fig — R3/1 cells were transfected with the indicated vectors, treated or not with an ADAM10 inhibitor (GI), and processed as described in Materials and Methods. Representative WB of cell lysates (50 μg) and supernatants of R3/1 cells. Because of the variable levels of cRAGEs in the supernatants of R3/1 cells, short and long exposures of the same membrane are shown. Beta-actin and red Ponceau staining were used as loading control for cell lysates and supernatants, respectively. (TIF) [file pone.0153832.s002.tif]

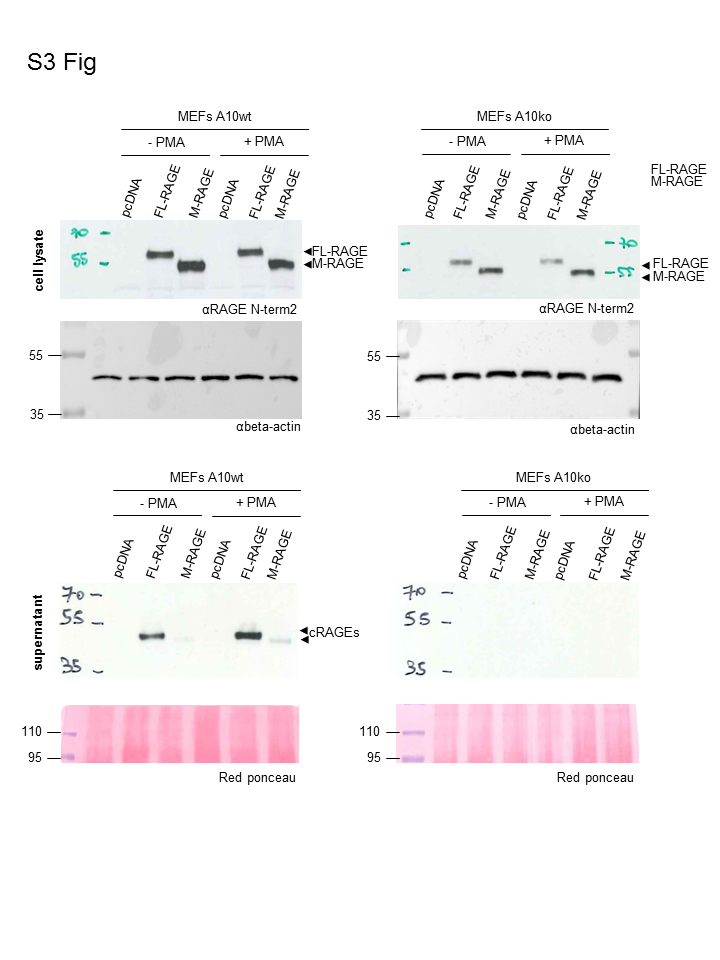

Supplement: S3 Fig — A10 ko and A10 wt MEFs were transfected with the indicated vectors, treated or not with PMA for 3 hours and processed as described in Materials and Methods. Representative WB of cell lysates (40 μg) and supernatants of MEFs. Beta-actin and red Ponceau staining were used as loading control for cell lysate and supernatant, respectively. (TIF) [file pone.0153832.s003.tif]
